# Carotid Artery Dissection as Initial Manifestation of Overlapping Sarcoidosis and Antiphospholipid Syndrome in a Young Woman

**DOI:** 10.1002/ccr3.73052

**Published:** 2026-06-25

**Authors:** Mohammad Ahmad Al‐Jazi, Anders Kildahl Johannsen, Anne Brink Behrndtz

**Affiliations:** ^1^ Department of Neurology, Physiotherapy and Occupational Therapy Gødstrup Hospital Herning Denmark; ^2^ NIDO, Center for Research and Education, Gødstrup Hospital Herning Denmark; ^3^ Department of Medicine Gødstrup Hospital Herning Denmark

**Keywords:** antiphospholipid syndrome, autoimmunity, carotid artery dissection, erythema nodosum, lupus anticoagulant, sarcoidosis, stroke

## Abstract

In young‐onset stroke with inflammatory features, consider systemic causes alongside vascular imaging. This case links cervical internal carotid artery dissection with coexisting sarcoidosis and antiphospholipid syndrome, highlighting the value of targeted work‐up and appropriate long‐term anticoagulation.

## Graphical abstract

Brain MRI demonstrating left hemispheric watershed infarctions and CTA showing high‐grade left internal carotid artery dissection with complete radiological resolution on follow‐up imaging.

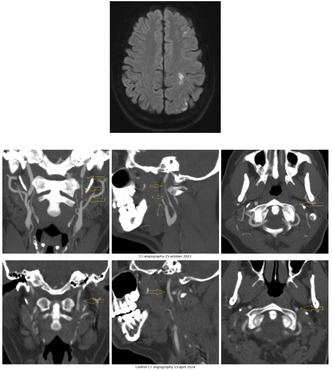

## 1 Introduction

Ischemic stroke in young adults accounts for 10%–15% of all strokes and often involves atypical etiologies such as arterial dissection, thrombophilia, and systemic autoimmune conditions [1]. Spontaneous cervical artery dissection (sCAD) is a common non‐atherosclerotic cause, frequently related to trauma, yet immune‐mediated mechanisms are increasingly implicated [2]. We report a rare case in which sarcoidosis and APS coexisted, leading to internal carotid artery dissection as the index vascular event in a young woman.

While sporadic cases of coexisting sarcoidosis and APS have been reported, no studies to date have proposed a mechanistic explanation for their combined role in arterial dissection.

## 2 Case History / Examination

A 37‐year‐old left‐handed woman with no known cardiovascular risk factors presented with acute right hemiparesis. Six days prior to presentation, she had developed multiple painful, poorly demarcated, tender erythematous–violaceous subcutaneous nodules on the posterior lower legs, clinically consistent with erythema nodosum (Figure 1). These systemic features were accompanied by low‐grade fever and an elevated C‐reactive protein (CRP) of up to 40 mg/L, indicating an inflammatory response that temporally preceded the focal neurological symptoms.

**FIGURE 1:**
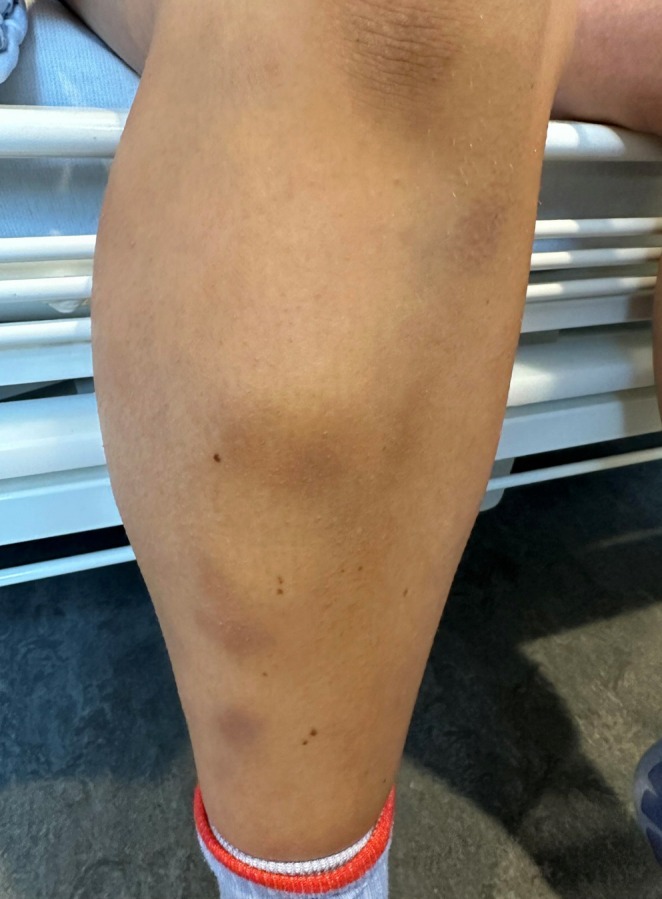
Erythema nodosum: Multiple tender erythematous–violaceous subcutaneous nodules on the posterior lower legs.

On examination, neurological deficits were confined to the right side, localizing the process to the left cerebral hemisphere. General examination corroborated the described cutaneous nodules over the posterior lower legs. No additional cardiovascular risk factors were identified on history.

## 3 Differential Diagnosis, Investigations, and Treatment

Given the acute focal neurological deficits, urgent exclusion of cerebrovascular pathology was paramount; in a young patient, a thorough etiologic workup for stroke is essential. Once stroke is established, the evaluation should prioritize young‐onset etiologies—particularly cervical arterial dissection, cardioembolic sources (structural or rhythm‐related), and inflammatory vasculopathies, while considering infectious mimics as appropriate. In parallel, the systemic inflammatory features warranted consideration of vasculitis and infectious etiologies.

Brain MRI demonstrated watershed infarctions in the left hemisphere (Figure 2). Computed tomography angiography (CTA) demonstrated ~90% near‐occlusion of the left internal carotid artery (ICA) at the C1 level with scant residual contrast opacification, most consistent with dissection (Figure 3). Time‐of‐flight magnetic resonance angiography (TOF‐MRA) of the neck vessels was also reviewed and supported an abnormality at the corresponding segment. An axial TOF image demonstrating a luminal abnormality consistent with dissection is provided (Figure 4).

**FIGURE 2:**
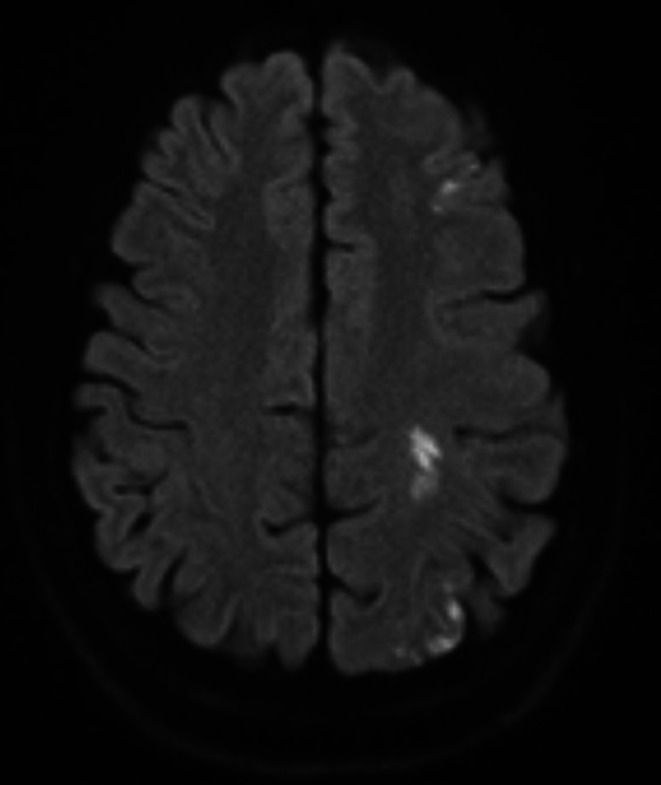
Brain magnetic resonance imaging (MRI) showing left‐hemispheric watershed infarctions involving the parietal and frontal regions, consistent with hypoperfusion secondary to carotid dissection.

**FIGURE 3:**
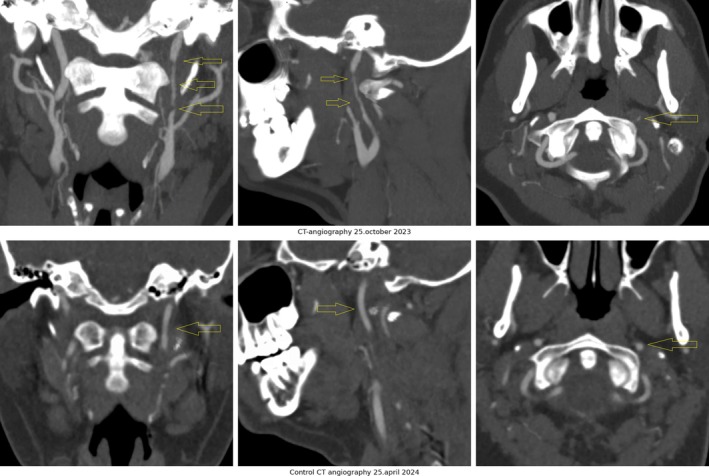
Computed tomography angiography (CTA) showing high‐grade left internal carotid artery (ICA) stenosis/near‐occlusion at the C1 level consistent with dissection (25 Oct 2023) with complete resolution on follow‐up CTA (April, 25 2024).

**FIGURE 4:**
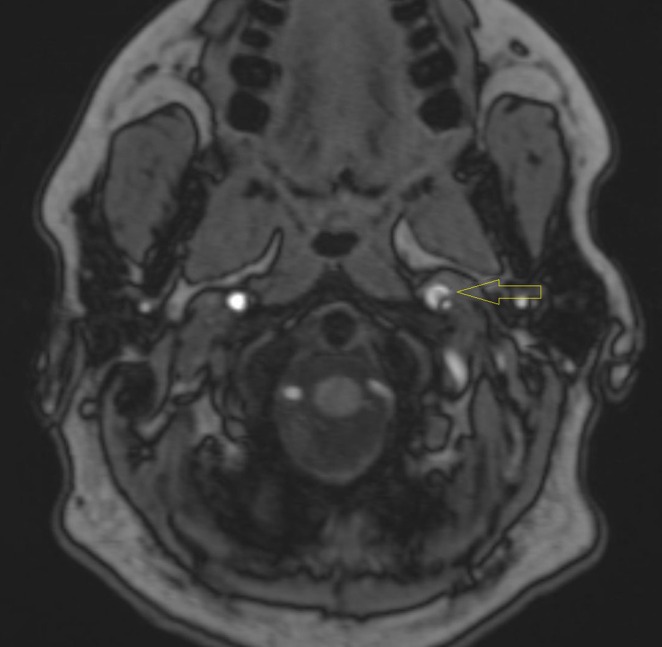
Axial time‐of‐flight magnetic resonance angiography (TOF‐MRA) of the neck vessels demonstrating an abnormal luminal signal within the left internal carotid artery (ICA) at the corresponding segment, supporting carotid artery dissection.

Color Doppler ultrasound of the extracranial and intracranial arteries showed a hemodynamically significant stenosis of the left ICA with markedly increased flow velocities; the otherwise normal appearance of the remaining vessels on duplex and CTA effectively excluded radiological evidence of cerebral vasculitis.

Given the systemic manifestations and skin findings, a high‐resolution computed tomography scan of the thorax, abdomen, and pelvis (CT‐TAP) was obtained, demonstrating symmetric bilateral hilar and mediastinal lymphadenopathy, which raised suspicion for sarcoidosis (Figure 5). Serum angiotensin‐converting enzyme (ACE) and soluble interleukin‐2 receptor (sIL‐2R) levels were within normal limits. Pulmonary function testing was largely unremarkable, except for a mildly reduced diffusing capacity for carbon monoxide (DLCO). The patient was referred to pulmonology, where sarcoidosis was diagnosed on clinical and radiological grounds; biopsy was deferred in accordance with current WASOG diagnostic criteria, given the classical features and absence of atypical findings [3]. In the context of fever and erythema nodosum, infectious mimics were considered. Urine and blood cultures were negative.

**FIGURE 5:**
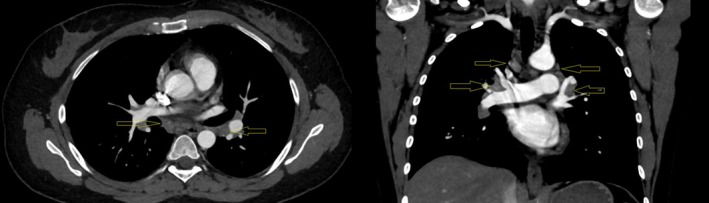
High‐resolution computed tomography (CT) of the thorax demonstrates symmetric bilateral hilar and mediastinal lymphadenopathy suggestive of sarcoidosis.

Incidental bilateral pulmonary emboli were identified on CT‐TAP. A thrombophilia work‐up confirmed persistent lupus anticoagulant positivity (LA1/LA2 ratio > 1.3) on two occasions more than 12 weeks apart, thereby establishing antiphospholipid syndrome (APS) according to the 2023 ACR/EULAR classification criteria [4]. To assess potential systemic involvement, transthoracic echocardiography and cardiac magnetic resonance imaging were performed; both were unremarkable, with no evidence of structural cardiac disease, pulmonary hypertension, or myocardial involvement. A dedicated transoesophageal echocardiography (TEE/TOE) bubble study was not performed.

The patient was initially treated with rivaroxaban and later transitioned to lifelong warfarin due to the combination of arterial and venous thrombotic events.

## 4 Outcome and Follow‐Up

The patient was discharged from the stroke unit to a specialized neurorehabilitation center and subsequently enrolled in cardiology and pulmonology outpatient follow‐up for ongoing clinical and biochemical monitoring. Six‐month follow‐up CTA demonstrated complete resolution of the left internal carotid artery dissection (Figure 3). Neurological function improved gradually, with mild residual weakness of the right hand.

On subsequent pulmonary follow‐up, no sarcoidosis‐directed therapy was initiated, as the patient reported no respiratory symptoms and lung function remained stable (dynamic parameters within the normal range and only mildly reduced diffusing capacity for carbon monoxide [DLCO]), with no paraclinical signs of sarcoidosis activity. The patient was therefore discharged from further routine sarcoidosis follow‐up, with advice to recontact the department in case of symptom recurrence.

## 5 Discussion

This case illustrates the rare combination of sarcoidosis and primary antiphospholipid syndrome (APS) leading to a stroke in a young woman. Arterial dissection is a well‐documented cause of ischemic stroke in younger adults [2], and inflammatory conditions may increase vascular fragility, predisposing patients to dissection [5]. When a stroke is accompanied by systemic symptoms such as fever, rash, or fatigue, underlying causes, as in this case, sarcoidosis, should be considered [6, 7]. Sarcoidosis is a multisystem granulomatous disorder that may present with pulmonary, cutaneous, or vascular involvement [2]. It involves the vasculature either through granulomatous infiltration or immune‐mediated mechanisms [8]. The diagnosis in this case was based on clinical presentation, characteristic radiological findings, and exclusion of differential diagnoses. A histological confirmation was not pursued, as the clinical and radiologic findings were considered sufficiently characteristic to support a presumptive diagnosis of sarcoidosis.

The subsequent benign pulmonary course without need for sarcoidosis‐directed therapy was consistent with the presumptive diagnosis. APS has been implicated in a notable proportion of strokes in young women, with some studies reporting rates up to 20% [9].

Co‐occurrence of sarcoidosis and APS has been previously reported in rare instances [10, 11]. However, to our knowledge, their concurrence with ICA dissection as the presenting event has only been reported in one case [12]. Warfarin remains the anticoagulant of choice for APS with arterial events, as direct oral anticoagulants (DOACs) have shown inferior protection in this context [13]. Multidisciplinary management was critical in this case, including neurology, pulmonology, cardiology, rheumatology, and hematology input.

Paradoxical embolism was considered a theoretical differential in the setting of concomitant venous thromboembolism (pulmonary emboli). However, the vascular imaging pattern was most consistent with a focal high‐grade left internal carotid artery (ICA) dissection with complete interval radiologic resolution on follow‐up computed tomography angiography (CTA). Transthoracic echocardiography (TTE) was unremarkable, and cardiac magnetic resonance imaging (CMR) showed normal biventricular function with no pathological late gadolinium enhancement (LGE) and no evidence of cardiac sarcoidosis. A dedicated transoesophageal echocardiography (TEE/TOE) bubble study was not performed; therefore, a very small shunt cannot be excluded with absolute certainty, but there was no supportive evidence for a paradoxical embolic mechanism in this case.

We propose that granulomatous inflammation in sarcoidosis may compromise vascular integrity through immune‐mediated endothelial activation, thereby increasing susceptibility to vascular injury in the presence of antiphospholipid antibodies [10, 12]. This case may exemplify a dual‐pathology mechanism, in which sarcoidosis and APS act synergistically to promote arterial dissection in predisposed individuals, consistent with a “two‐hit” model previously described in thrombo‐inflammatory vascular disease [14].

## 6 Conclusion

This case describes the rare coexistence of sarcoidosis and primary antiphospholipid syndrome (APS), with internal carotid artery dissection as the initial vascular event. The case suggests the etiology may involve an overlap between granulomatous and thrombo‐inflammatory autoimmunity.

Young patients presenting with stroke should be thoroughly examined, and evaluation for systemic inflammatory or autoimmune conditions should be considered in addition to the standard workup. This case emphasizes the importance of multidisciplinary assessment and long‐term follow‐up. Awareness of this association is essential for timely diagnosis and an appropriate anticoagulation strategy.

## Author Contributions

**Mohammad Ahmad Al‐Jazi:** conceptualization, data curation, formal analysis, investigation, writing – original draft, writing – review and editing. **Anders Kildahl Johannsen:** validation, review and editing. **Anne Brink Behrndtz:** supervision, validation, review and editing.

## Funding

The authors have nothing to report.

## Ethics Statement

Ethics committee approval was not required for the publication of this single anonymized case report in accordance with local institutional policy.

## Consent

Written informed consent was obtained from the patient for publication of this case report and accompanying images. The patient reviewed the manuscript and all images to be included prior to publication and signed a consent‐to‐disclose/publication form.

## Data Availability

Data sharing does not apply to this article, as no datasets were generated or analyzed during the current study.
